# Chromosomal and Molecular Diversity in the *Simulium ornatum* Group (Diptera: Simuliidae) in the Western Tian Shan Range of Central Asia

**DOI:** 10.3390/insects12090817

**Published:** 2021-09-12

**Authors:** Peter H. Adler, Doreen Werner, Helge Kampen

**Affiliations:** 1 Department of Plant and Environmental Sciences, Clemson University, Clemson, SC 29634-0310, USA; 2 Leibniz Centre for Agricultural Landscape Research, 15374 Müncheberg, Germany; Doreen.Werner@zalf.de; 3 Friedrich-Loeffler-Institut, Federal Research Institute for Animal Health, 17493 Greifswald, Germany; Helge.Kampen@fli.de

**Keywords:** aquatic insects, black flies, CO1 sequences, Kyrgyzstan, polytene chromosomes, *Simulium ferganicum*, *Simulium mesasiaticum*, taxonomy

## Abstract

**Simple Summary:**

Members of the *Simulium ornatum* group are among the most abundant and widely distributed black flies in the Palearctic region. We investigated the chromosomal, molecular, and morphological diversity of this group across the varied landscape of Kyrgyzstan. Morphology and chromosomal band patterns suggested the presence of one or two species, whereas mitochondrial DNA sequences indicated up to three species. We linked the name *Simulium mesasiaticum* with cytologically undifferentiated sex chromosomes, one of three mitochondrial DNA clusters, and higher elevations. We tentatively associated the name *Simulium ferganicum* with differentiated sex chromosomes, a second DNA cluster, and lower elevations. One Kyrgyz larva in a third DNA cluster could not be linked to a formal species name. The analyses also indicated that *Simulium ornatum*, the namesake of the entire group, does not occur in Kyrgyzstan despite previous records. The results demonstrate that using only one source of data does not provide a complete picture of the biodiversity and integrated taxonomy is often necessary for species resolution in the family Simuliidae.

**Abstract:**

By any measure, such as abundance, species diversity or geographic range, the *Simulium ornatum* species group is one of the most successful Palearctic taxa of black flies. To explore potential diversity in this group in the Tian Shan range of Central Asia, we focused on Kyrgyzstan, in which three nominal morphospecies have been recorded. Among our samples, we morphologically identified *S. mesasiaticum* Rubtsov and a second possible species tentatively identified as *S. ferganicum* Rubtsov. By analyzing banding patterns of the larval polytene chromosomes, we discovered two fixed inversions, two sex-linked rearrangements, and 19 autosomal rearrangements, including supernumerary B chromosomes. The chromosomal data indicate minimal diversity of only one or two species across the surveyed area of nearly 50,000 km^2^. Mitochondrial DNA (CO1) sequences fell into three distinct clusters, possibly representing separate species. The chromosomal, molecular, and morphological data indicate that Kyrgyz populations are unique within the *S. ornatum* group, but the data sets are not entirely congruent. Thus, reconciling data sets and assigning existing names is tentative. *Simulium mesasiaticum* is linked with undifferentiated sex chromosomes, one of the three CO1 clades, and higher elevations, whereas *S. ferganicum* is tenuously associated with differentiated sex chromosomes, a separate CO1 clade, and lower elevations. These associations leave one Kyrgyz larva, which is in a third CO1 clade, unlinked to a formal species name. Our analyses also indicate that *S. ornatum* Meigen *sensu stricto*, contrary to previous reports, does not occur in Kyrgyzstan and should be deleted from the country’s faunal list.

## 1. Introduction

The success of a group of insects can be judged, relative to taxa of equal rank, on factors such as species diversity, relative abundance, and breadth of geographic distribution. Accordingly, *Simulium* would be considered the most successful subgenus of the Simuliidae. Accounting for nearly 23% of the family’s species, it is the largest and most widely distributed of the 47 subgenera [1]. Of the 33 species groups in the subgenus *Simulium*, the *Simulium* (*S.*) *ornatum* group is one of the most widely distributed in the Palearctic region. Its members are among the most abundant and commonly encountered black flies and have fared particularly well in areas degraded by land development, livestock grazing, and pollution [2].

Assessing species diversity depends on recognizing all species, often a challenging task given the potential for cryptic species [3], although indicators such as abundance and the broad habitat range of a nominal species can signal their presence [4]. Chromosomal and molecular analyses have uncovered an abundance of hidden diversity in the Simuliidae [5,6,7]. The *Simulium ornatum* group, consisting of 24 species [1], is not the largest species group in the subgenus, a position held by the *S. tuberosum* group with 69 nominal species. The latter group, however, has received intense scrutiny for cryptic species [8,9,10,11], whereas the former has received scant screening. Geographically limited chromosomal analyses nonetheless suggest that diversity in the *S. ornatum* group is underappreciated [12].

We used the banding patterns in the giant polytene chromosomes of the larval silk glands to investigate the diversity in the *S. ornatum* group in the western Tian Shan region, an immense mountain system in Central Asia. We also performed a mitochondrial CO1 (cytochrome c oxidase subunit 1) analysis. At least five nominal species in the group have been recorded from this area [13,14], suggesting a rich fauna for the group and the potential for discovering cryptic diversity. The deeply dissected topography of the Tian Shan region suggests opportunities for isolation and fragmentation of populations, further supporting the notion that diversification of the group might have occurred. We focused on the Tian Shan region of Kyrgyzstan, which is in the heartland of the Central Asian *S. ornatum* group, where three formally named morphospecies have been reported.

## 2. Materials and Methods

### 2.1. Sampling and Identification

Larval and pupal simuliids were collected from all available substrates, primarily trailing vegetation and stones, in 99 streams throughout central Kyrgyzstan from 17 June to 7 July 2015. Larvae were fixed in 1:3 acetic ethanol, which was refreshed at least once within 30 min of collecting and again within 8 h. A corresponding collection of larvae at each site was also fixed in 95% ethanol. To aid identification, pupae (when available) were placed in Petri dishes with moist filter paper and reared in the field to adults. Nuisance black flies swarming about the collectors were taken with an aerial net, as were incidental adults that entered our tents at the campsites in the evenings. All material belonging to the *S. ornatum* group was sorted from our samples. A total of 6 females and 6 males reared from pupae (site 52) and two females collected inside the tents (near sites 18 and 34) were chemically dried with hexamethyldisilazane (Alfa Aesar, Ward Hill, MA, USA) [15] and pinned; pupal exuviae and cocoons in glycerin vials were pinned beneath reared adults. We morphologically identified all material to species using the dichotomous keys of Rubtsov [13]. Pinned adults, pupae, and larvae (after transferal from acetic acid to ethanol) were deposited in the Clemson University Arthropod Collection.

### 2.2. Chromosomal Conventions and Procedures

The expanded posterior segments of the abdomen of the antepenultimate through ultimate larval instars (before pharate pupal formation) of the *S. ornatum* group were removed, opened ventrally with fine needles, and stained using the Feulgen procedure [16]. Polytene chromosomes in silk-gland tissue, plus one gonad for gender determination, were squashed on a microscope slide in a drop of 50% acetic acid and interpreted and photographed with bright-field microscopy under oil immersion. Pale and dark larvae at each site were analyzed separately to test for an association with sex.

We used the universal chromosomal conventions for the Simuliidae [8]. Specifically, the three submetacentric chromosomes were numbered I, II, and III in order of decreasing length, with a short (S) and long (L) arm on either side of each centromere. The entire complement was divided into 100 sections corresponding to those of the *Simulium* subgeneric standard [16,17]. We identified the sex chromosomes when they were microscopically detectable. In the Simuliidae, any of the three chromosomes can function as the sex chromosome. The banding patterns of the X and Y can be microscopically identical (X_0_Y_0_) or differentially associated with rearrangements (e.g., inversions) that are expressed heterozygously in the heterogametic sex, typically the male (e.g., X_0_Y_1_, X_1_Y_0_, and X_1_Y_1_). Sex-chromosome polymorphism with the linkage of different rearrangements among individuals (e.g., X_1_Y_1_, X_2_Y_1_, and X_3_Y_2_) is not uncommon in a population.

Chromosomal banding patterns were compared against the *Simulium* subgeneric standard maps by Rothfels et al. [17] for chromosome arms IS, IL, IIL, and IIIS, and by Adler et al. [16] for IIS and IIIL. Images of diagnostic sequences were made with a Jenoptik ProgRes^®^ SpeedXT Core 5 digital camera (JENOPTIK Optical Systems, Huntsville, AL, USA) on a BH-2 Olympus microscope (Olympus Corporation, Center Valley, PA, USA) and chromosomal maps were prepared with Adobe^®^ PhotoShop^®^ Elements 8 (Adobe Systems Incorporated, San Jose, CA, USA). Chromosomal mapping procedures and terminology follow those of Adler et al. [9,16]. All rearrangements were mapped. Only fixed inversions were italicized. Y-linked rearrangements were shown with a dashed bracket on the chromosome maps. A number of different labelling systems are in place for inversions [18]. However, recent practice, as followed here, has been to number inversions sequentially in order of discovery within a taxon, typically within each species group for the subgenus *Simulium*, e.g. [9], although inversions shared among species groups are given the same number (e.g., *IIIL-1*). This paper represents a series of ongoing investigations of the *S. ornatum* group and therefore complicates the inversion numbering. We therefore began numbering new inversions discovered in this study with a sufficiently high number to avoid duplication of numbers in other group members currently under study, while still allowing for future continuous numbering of inversions discovered in the group. Each heteroband (hb) was named for its chromosome arm and section number (e.g., IIIS hb81), although amplified telomere bands (e.g., IIIS hb telo) were explicitly indicated. Each deleted band (de) was similarly named (e.g., IIIS de79). Each secondary nucleolar organizer (2ºNO) was labeled.

### 2.3. Molecular Procedures

Fourth-instar larvae that could be confidently identified morphologically as members of the *S. ornatum* group were taken from all ethanol samples that contained the group. These larvae (*n* = 18) were subjected to mitochondrial CO1 barcoding [19]. DNA extracted from about three abdominal segments of each larva was PCR-amplified, sequenced, and analyzed following procedures described by Kampen et al. [20].

A neighbor-joining tree was constructed using the Geneious Tree Builder integrated in Geneious Prime 2020.0.1 (genetic distance model HKY) based on partial CO1 gene sequences aligned by MAFFT version 7.388. Sequences were obtained from our Kyrgyz larvae of the *S. ornatum* group, associated black flies at some Kyrgyz sites, and selected reference species from GenBank, which reflect species used in other CO1 studies of the *S. ornatum* group [21,22]. *Aedes japonicus* (Diptera: Culicidae) was defined as an outgroup.

With the exception of two specimens that were not assignable to a species or species group (D19-33/2 and D19-33/3), all CO1 sequences generated in this study were deposited in GenBank (accession numbers MW741560–MW741562 and MW748276–MW748293).

## 3. Results

### 3.1. Sampling Sites

**Figure 1:**
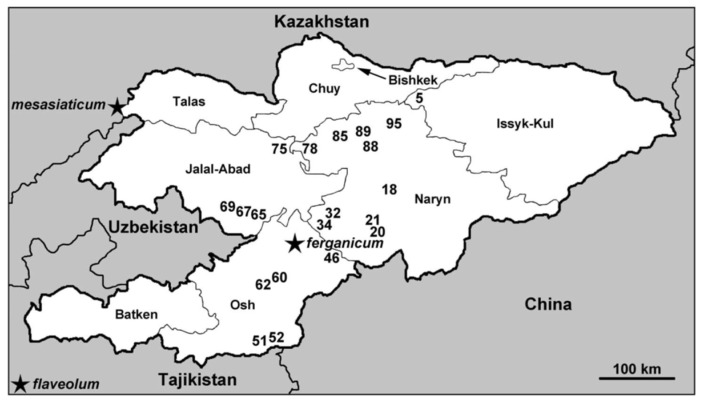
Map of Kyrgyzstan showing sites at which the *Simulium ornatum* group was collected, June–July 2015. Regions are labeled, details corresponding to site numbers are in Table 1, and type localities of three nominal species of the group are indicated with a star.

Of 99 sampling sites in Kyrgyzstan, 20 sites within an area of roughly 50,000 km^2^ had larvae of the *S. ornatum* group (Table 1, Figure 1). The streams harboring the group trickled through alpine meadows, flowed through arid valleys, or tumbled down rocky mountainsides. They were cool (3–20 °C) and 0.15–20 m wide, with open canopies. Other than females and males reared from pupae, the only additional adults of the *S. ornatum* group that we found were two females inside our tents. Females were absent from nuisance swarms around people, suggesting that humans are typically not used as blood hosts.

### 3.2. Morphology

We morphologically identified all reared and incidentally collected adults as *Simulium mesasiaticum* Rubtsov. Larvae and pupae of *S. mesasiaticum* are unknown. Pupal exuviae associated with reared adults that we identified as *S. mesasiaticum*, plus all other pupae and mature larvae in our samples, keyed to *S. ferganicum* Rubtsov, suggesting that the immature stages of the two species are similar. *Simulium ferganicum* might be present in our samples but we did not find adults that key to this species and if the immatures of the two species are not separable by conventional means, morphological confirmation of *S. ferganicum* in our material is not possible. The degree of larval pigmentation in our samples did not correspond with sex but rather with the substrate from which larvae were collected; pale larvae generally were taken from vegetation and dark larvae from stones.

### 3.3. Chromosomes

Of 341 larvae of the *S. ornatum* group collected in Kyrgyzstan, 157 were prepared for chromosomal analysis, of which 139 (88.5%) could be read entirely and were included in analyses (Table 2). Most sites had some larvae too small for analysis and at sites 18 and 21, all larvae were too small.

**Figure 2:**
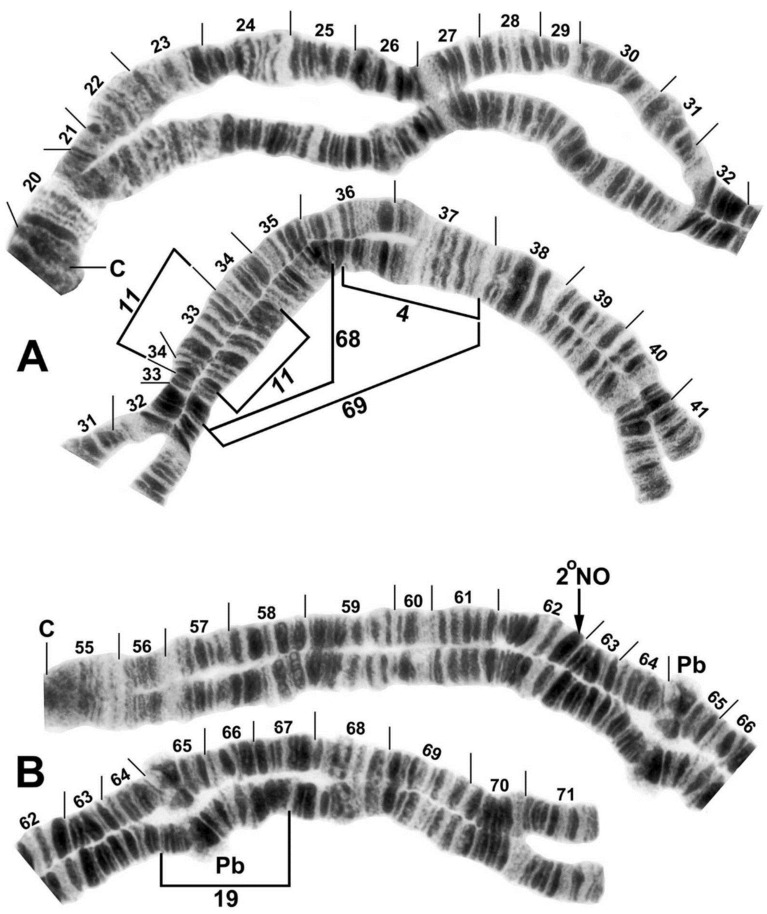
Chromosomes IL and IIL of the *Simulium ornatum* group from Kyrgyzstan; C, centromere. (**A**) IL showing the *IL-11* sequence; photocomposite of female larvae from site 21 (sections 20–31) and site 5 (sections 32–41). Breakpoints of polymorphic inversions IL-4, IL-68, and IL-69 are indicated by brackets. (**B**) IIL showing the standard sequence of the subgenus *Simulium*; photocomposite of a male larva from site 85 (sections 55–62 proximal) and female larva from site 65 (sections 62 distal–71). Breakpoints of the polymorphic inversion IIL-19 are indicated by a bracket. Pb, parabalbiani; 2°NO, location of secondary nucleolar organizer (within the band).

**Figure 3:**
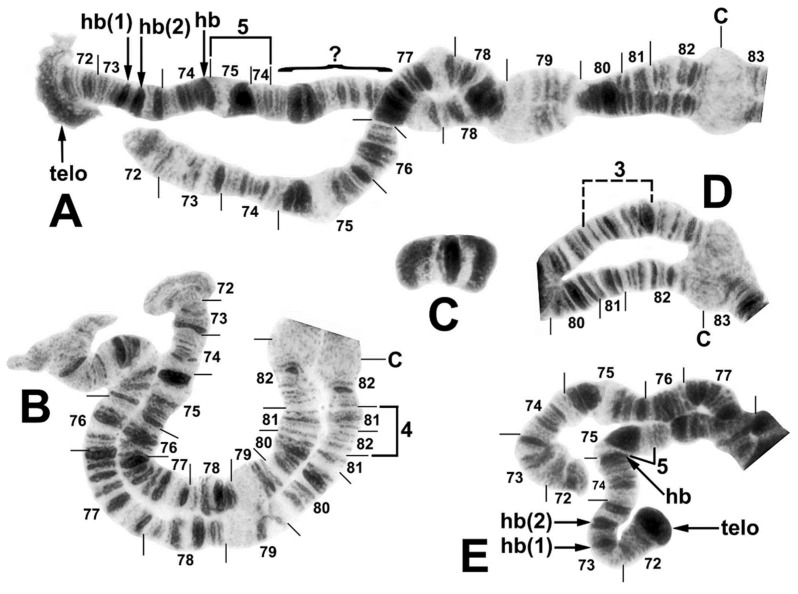
Chromosome IIIS (male larvae) and supernumerary B chromosome (female larva) of the *Simulium ornatum* group from Kyrgyzstan; C, centromere. (**A**) IIIS heterozygous for repatterning (site 5). The repatterned homologue shows inversion IIIS-5, a flared and amplified telomere (telo), and 3 heterobands (hb) in sections 73 and 74, of which the middle band is a doublet; sections 75–76 could not be reconciled with the standard homologue and are indicated with a bracket and query mark. (**B**) IIIS heterozygous for IIIS-4 (site 85), bracketed; the upper homologue shows the standard sequence for the subgenus *Simulium*. The distal ends of both homologues are flared. (**C**) Supernumerary B chromosome (site 21). (**D**) Base of IIIS heterozygous for Y-linked IIIS-3; the inversion is bracketed (site 34). (**E**) End of IIIS heterozygous for repatterning (site 5); the features are the same as in (**A**), although the amplified telomere is compact.

**Figure 4:**
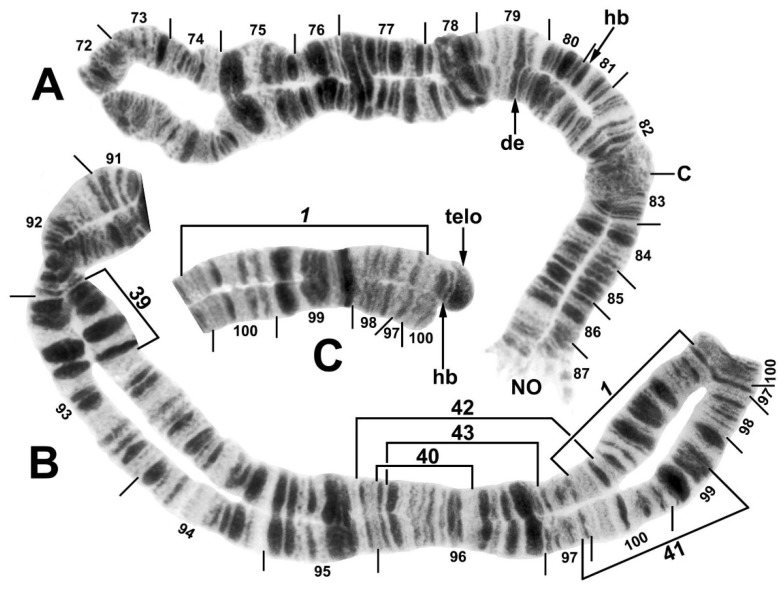
Chromosome III of the *Simulium ornatum* group from Kyrgyzstan. (**A**) IIIL base and IIIS heterozygous for a single band deletion (de), indicated with an arrow pointing to where the missing band should be (female larva, site 60). Location of a heteroband (hb, not shown) is indicated with an arrow; C, centromere; NO, primary nucleolar organizer. (**B**) IIIL (sections 91–100) showing the *IIIL-1* sequence (female larva, site 34). Breakpoints of polymorphic inversions IIIL-39, IIIL-40, IIIL-41, IIIL-42, and IIIL-43 are indicated by brackets. (**C**) IIIL end showing the *IIIL-1* sequence and heterozygous configuration for an amplified telomere (telo), as well as its associated subterminal band (hb) in section 100, both indicated with arrows (composite male larva, site 5).

The haploid complement of three chromosomes had up to 65% unpairing of homologues (Figure 2A). The centromere (C) regions were expanded, particularly the CI region (Figure 2A), and centromere bands CII and CIII were diffuse and weakly stained (Figure 2B, Figure 3A,B,D, and Figure 4A); the CI band was variably stained and distinct (Figure 2A). A chromocenter and ectopic pairing of the centromere bands were lacking, although ectopic pairing occurred sporadically in other areas of the complement. The primary nucleolar organizer was at the junction of sections 87 and 88 in the standard location for the subgenus *Simulium* (Figure 4A). The distal ends of IIIS were either flared (Figure 3B), perhaps indicating gene expression, or compact (Figure 4A).

All Kyrgyz samples differed from the subgeneric standard by two fixed inversions, namely *IL-11* (Figure 2A) and *IIIL-1* (Figure 4B). Nineteen autosomal polymorphisms were found, distributed as eleven inversions (Figure 2A,B, Figure 3A,B, Figure 4B), six band rearrangements (Figure 3A,E and Figure 4A,C), one secondary nucleolar organizer (Figure 2B), and supernumerary B chromosomes (Figure 3C). With the exception of IL-4 (Figure 2A), these polymorphisms were in low frequencies (Table 2). IL-4 was found only in a small cluster of sites (88 and 95) in the northeastern portion of our surveyed area and at site 88, it had a frequency of 0.35. IIIL hb100 + telo (Figure 4C) was found at four sites. The mean number of the heterozygous inversions per larva was low (0.13).

The sex chromosomes typically were microscopically undifferentiated (X_0_Y_0_), although in the west-central surveyed area, alternative sequences in IIIS were linked to the genetic X and Y chromosomes (Table 2). Thus, 75.4% of all males were X_0_Y_0_, whereas 24.6% had IIIS-3 (X_0_Y_1_) (Figure 3D). IIIS-3 had its highest frequency at site 65, where eight of ten male larvae (but zero of twelve female larvae) carried it. One sex-exceptional female was heterozygous for IIIS-3 at site 67. Females were predominantly X_0_X_0_ but 12.8% had a mildly amplified heteroband (IIIS hb81) that was absent in males (Figure 4A), typically in the heterozygous condition (X_0_X_1_) but homozygous (X_1_X_1_) in one female. IIIS-4 (Figure 3B) and IIIS de79 (Figure 4A), as single-occurrence heterozygotes, also might be sex-linked but not enough evidence is available; they were found at sites with no other IIIS rearrangements and are treated as autosomal in Table 2. IIIS hb telo was heterozygous in two female and two male larvae at site 88, suggesting that it was not sex-linked. It also was present in one male larva at site 5. The enhanced telomere typically was flared and disorganized (Figure 3A). However, in the male from site 5, both the flared condition (Figure 3A) and a compact, darkly staining condition (IIIS hb telo) (Figure 3E) were found in different nuclei. IIIS hb telo in this same male and in one male from site 88 was associated with a complex repatterning of the distal half of the arm, involving at least one inversion (IIIS-5) and several band amplifications (IIIS hb73(1), IIIS hb73(2), IIIS hb74, and IIIS hb telo) (Figure 3A,E).

### 3.4. CO1 Sequences

**Figure 5:**
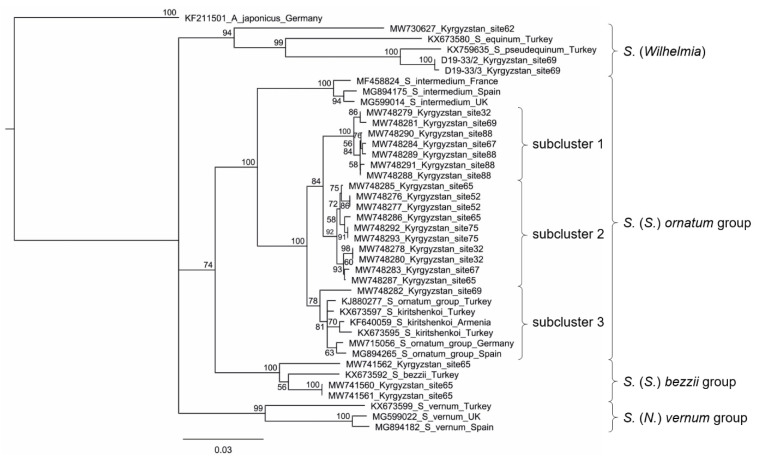
Partial CO1 sequence-based neighbor-joining tree showing the *Simulium ornatum* group, associated black flies in our samples, and selected reference species from GenBank. *Aedes japonicus* (Diptera: Culicidae) was used as an outgroup. Statistical support of 1000 bootstraps is indicated at the nodes. The scale bar represents the number of substitutions per site. D19-33/2 and D19-33/3 are laboratory identification numbers; these sequences could not be assigned to a species or species group and therefore could not be entered into GenBank.

The mitochondrial DNA barcode tree shows four clusters in the *S. ornatum* group (Figure 5). *Simulium intermedium* Roubaud is in a distinct clade sister to all other specimens of the group. *Simulium kiritshenkoi* Rubtsov and European members of the *S. ornatum* group form a cluster without resolution, which is sister to one Kyrgyz specimen in the same cluster. All other Kyrgyz specimens of the *S. ornatum* group are clustered in two clades of seven and ten specimens each.

CO1 sequences (709 bp including primer annealing sites) varied within subclusters 1 and 2 of the *S. ornatum* group by up to ca. 1.3% (9 bp), whereas in the less homogeneous subcluster 3, which—in addition to one Kyrgyz specimen—contained sequences derived from GenBank, they varied by about 2.4% (17 bp) (Figure 5). In contrast, sequence differences among the three clusters ranged from about 2.1 to 4.1% (15–29 bp).

## 4. Discussion

Integrated studies are often needed for the resolution of taxonomic problems in the Simuliidae and ideally involve morphological, chromosomal, and molecular analyses [23,24], sometimes coupled with ecological data [25]. Even still, these data sets are not always in agreement or equal in their ability to resolve similar species [26].

Morphological identifications of our Kyrgyz material indicated the presence of *S. mesasiaticum* and suggested the possible presence of *S. ferganicum*. Several caveats, however, are in order. The diagnostic characters for adults are mostly minor differences in color [13], which in the Simuliidae can vary with elevation, season, and temperature [27,28,29]. Diagnostic characteristics for pupae of species in the *S. ornatum* group [13], such as the length of the gill petioles, are known to vary intraspecifically in many black flies [8]. Conventional identification of larvae of the *S. ornatum* group [13] also relies to a considerable extent on spurious characters that are environmentally influenced, such as the number of primary rays in the labral fan and number of hooklets in the posterior circlet [30,31,32]. Larval color can be diagnostic for some species, related to gender in others [8], or, as with the larvae in our samples of the *S. ornatum* group, correlated with the substrate on which they are found [33].

The *S. ornatum* group in Kyrgyzstan is chromosomally unique among all the studied nominal members of the group. Kyrgyz populations are derived from the subgeneric standard banding sequence by a mere two fixed inversions. *IL-11* is found widely among members of the *S. ornatum* group and *IIIL-1* is fixed in a number of species groups [26,34]. Of the polymorphic rearrangements in Kyrgyz larvae, three are found in other species of the *S. ornatum* group. IL-4 occurs in multiple members of the group throughout much of the western Palearctic region and we have found the enhanced IIIL telomeric band in several cytoforms of the *S. ornatum* group in eastern Europe and western Asia. B chromosomes occur sporadically in group members and the Kyrgyz B shows homology with that of *S. ornatum* Meigen *sensu stricto* [35].

Chromosomally, the general features and fixed-banding sequences are homogeneous across all sites. The sex chromosomes, however, are expressed in undifferentiated (X_0_, Y_0_) and differentiated (X_1_, Y_1_) configurations. The sex chromosomes of most individuals (82%) are microscopically undifferentiated but some individuals express alternative sex-chromosome sequences (IIIS-3 in males and IIIS hb81 in females). Some or all polymorphisms in IIIS might be sex-linked but we have adequate evidence to implicate only two (IIIS-3 and IIIS hb81). Species that lack fixed differences but have different sex chromosomes are common in the Simuliidae [36,37,38]. Thus, we are open to the possibility that individuals with differentiated sex chromosomes might represent a species separate from individuals with undifferentiated sex chromosomes, although the existing evidence is weak and the more conservative view is that they represent sex-chromosome polymorphism in a single species.

Of the remaining polymorphisms (i.e., all except sex-linked IIIS-3 and IIIS hb81), none clearly indicate distinct groups of larvae, although some (e.g., IL-4 and B chromosomes) show slight regional localization. Only seven (IL-4, IIIL hb100 + telo, and the apparent linkage set of IIIS-5, IIIS hb73(1), IIIS hb(2), IIIS hb74, and IIIS hb telo) are shared across Kyrgyz sites. If sample sizes had been larger, however, perhaps more polymorphisms would have been shared. The occurrence of B chromosomes only at one of our two highest elevation sites (>3200 m), without other polymorphisms, might reflect the peripheral nature of high-elevation habitats [26]. B chromosomes could provide an extra measure of variability [39]. In some plants, they can increase recombination in the primary (A) chromosomal complement and introduce new genes [40]. If similarly disposed in simuliids, they could introduce the needed variation for populations in peripheral habitats.

Similar to the chromosomal results, the CO1 data indicate that Kyrgyz populations of the *S. ornatum* group are unique. CO1 sequences of Kyrgyz specimens in the group fall into three subclusters. Although CO1 sequence diversity can be minute between species and interspecific diversity might overlap with intraspecific diversity, depending on the taxonomic group [41], the divergence among the three subclusters of the *S. ornatum* group from Kyrgyzstan suggests multiple species. Additional genetic analyses targeting the complete mitochondrial genome or nuclear genes are required to test this hypothesis. CO1 sequences of larvae from three sites are represented in each of two subclusters, indicating habitat overlap of molecular forms.

Ecologically, our chromosomal and molecular analyses include material from a broad elevational range (1098–3270 m) and three orders of magnitude in stream width (0.15 to 20 m wide). *Simulium ferganicum* has previously been reported from cold to very warm (7–29 °C) streams and rivers, nearly always with dense grassy vegetation, at elevations of 1200–1800 m in the valleys and foothills of the western Tian Shan [14]. The morphologically similar species *S. flaveolum* Rubtsov and *S. mesasiaticum* have been reported at higher elevations (2200–2500 m) in the meadow–forest areas of Central Asia [14].

Individuals with differentiated sex chromosomes were nested around the type locality of *S. ferganicum*, whereas those with cytologically undifferentiated sex chromosomes were more peripheral and included populations from among the highest sampled elevations where we morphologically identified *S. mesasiaticum*. Subcluster 2 in the mitochondrial DNA barcode tree consists of specimens collected at higher elevations (1743–3246 m) and includes the site from which we reared adults morphologically identified as *S. mesasiaticum*. Accordingly, a connection can be made among *S. mesasiaticum*, higher elevations, undifferentiated sex chromosomes, and subcluster 2 in the mt-DNA tree. A far weaker connection can be drawn among *S. ferganicum*, lower elevations (1098–2185 m), differentiated sex chromosomes, and subcluster 1. These connections, however, leave the status of the single Kyrgyz larva in subcluster 3 unknown.

We had expected greater chromosomal diversity in our samples given that the type localities of three nominal species of the *S. ornatum* group are in or near Kyrgyzstan. The type locality of *S. ferganicum* is in the heart of our surveyed area, that of *S. mesasiaticum* is in Kazakhstan about 165 km from our nearest collection site (69), and that of *S. flaveolum* is in Tajikistan near the border with Kyrgyzstan about 400 km from our nearest collection site (51). *Simulium flaveolum* also has been reported from Kyrgyzstan near Bishkek [13]. Furthermore, given the high frequency of cryptic species in the Simuliidae [6,42] and the number of larvae (139) and sites (18) that we examined, we expected multiple cytoforms. For instance, chromosomal study of the morphospecies *S. rufibasis* ‘B’ in northern Vietnam (Lao Cai Province), an area about one-third the size of our Kyrgyz sampling area, revealed five species among 137 larvae at six sites [9].

Sampling at the type localities of *S. flaveolum* and *S. mesasiaticum* is needed. The type localities are, however, somewhat vague and based on adults that could have flown some distance from their natal sites. *Simulium flaveolum* was described by Rubtsov in 1940 [43] as a variety of *S. ornatum* and *S. mesasiaticum* was described by Rubtsov in 1947 [44]. Lectotype females of *S. flaveolum* and *S. mesasiaticum* were designated by Yankovsky [45]. That of *S. flaveolum*, however, is invalid because it is not an original syntype, having been collected four years after the original type series. Rubtsov’s ([27], p. 531) brief original description of *S. flaveolum* did not give the type locality, although he later stated that it is the Kondarinka River in Tadjikistan [13].

The type locality of *S. ferganicum*, a species described by Rubtsov in 1940 [27], also carries some confusion. Rubtsov [27] gave the type locality as Mikhailovka in the Kugart range of the Fergana region, and the holotype male in the Zoological Institute, St. Petersburg, Russia, bears a label reading (in Cyrillic) “Mikhailovka, valley of river Kugart, Fergansk region”. Rubtsov [13], however, stated that the type specimen is from the “environs of Frunze”, the old name for Bishkek, which is about 210 km to the northeast of Mikhailovka. We recognize the legitimate type locality as the Kugart River valley of the Jalal-Abad region and assume that Rubtsov [13] took the liberty of giving the capital city area (“Frunze”) as a general indicator of the specific type locality. Mikhailovka is a hydropost where the Kugart River debouches from the mountains about 50 km from the river’s mouth [46]. Thus, we have collections (sites 32, 34, 65, and 67) from within 35–50 km of the type locality.

Rubtsov [13] and Konurbaev [14] claimed that *S. ornatum s. s.* also inhabits Central Asia, including Kyrgyzstan. However, its type locality (Germany) is well over 4000 km from Kyrgyzstan and no chromosomal evidence [12,47,48] supports the presence of *S. ornatum s. s.* in Kyrgyzstan. We therefore expunge *S. ornatum s. s.* from the Kyrgyz faunal list.

One additional nominal species, *S. deserticola*, might be expected in Kyrgyzstan. Although it has never been recorded from Kyrgyzstan and we found no evidence of its presence in our material, there are records for Tajikistan and Uzbekistan. Its original description is based on material from western Mongolia [43], about 1100 km from Kyrgyzstan. The larval chromosomes of a small sample (*n* = 2) of Mongolian larvae show little similarity with those of the Kyrgyz larvae, other than the presence of the taxonomically and geographically widespread IL-4 and *IIIL-1*. We do not know if these Mongolian larvae represent *S. deserticola*; the diagnostic feature, a long stalk for the ventral pair of pupal gill filaments [13], could not be evaluated in the two immature larvae. The Mongolian material is from 1140 km east of the original type locality of *S. deserticola* and about 2500 km northeast of our Kyrgyz sampling sites. Despite extreme allopatry, we suspect that the chromosomal differences between the Mongolian and Kyrgyz samples represent different species. The situation is further complicated, however, because the original type material from Mongolia was lost and Yankovsky [45] designated a neotype from Dushanbe, Tajikistan, about 400 km to the east of our nearest Kyrgyz collections. Sixteen years after the original description of *S. deserticola*, Rubtsov [13] gave its distribution as Tajikistan without mentioning Mongolia.

The possibility exists that we missed some diversity by not sampling in the easternmost and westernmost parts of Kyrgyzstan or by not sampling before mid-June or after early July. However, we collected near sites where the nominal species previously recorded in Kyrgyzstan [13,14,43,44] had been collected. In addition, all members of the *S. ornatum* group are multivoltine. Thus, we suspect that we would have encountered all nominal species of the group during our sampling period (mid-June to early July), particularly given the 1500-m elevational gradient that we covered. Overall, we consider the probability rather low for having missed chromosomally distinct species but recognize that more samples for molecular analysis might have revealed additional diversity.

## 5. Conclusions

Taken as a whole, the chromosomal, molecular, and morphological data sets indicate one to three species in the *S. ornatum* group in the surveyed area of Kyrgyzstan. Tenuous connections can be drawn among entities in the three data sets. Accordingly, cytologically undifferentiated sex chromosomes and a distinct subcluster in the CO1 tree are linked to *S. mesasiaticum*, whereas differentiated sex chromosomes and another subcluster tie less confidently to *S. ferganicum*, leaving the sole Kyrgyz larva, which is in a third subcluster, unassociated with a name. No evidence supports the presence of *S. ornatum s. s.* in Kyrgyzstan and it is removed from the country’s faunal list. Overall, the investigation demonstrates that data from only one source, whether chromosomal, molecular, or morphological, cannot always provide taxonomic resolution and that integrated studies are therefore needed.

## Figures and Tables

**Table 1:** Collection information for the *Simulium ornatum* group in Kyrgyzstan, June–July 2015, with numbers of larvae prepared chromosomally and sequenced for the CO1 gene.

Site	Location ^1^ (Stream Width)	Latitude and Longitude	Elevation (m Above Sea Level)	Date	Larvae Collected: Chromosome Preparations ^2^	CO1 Sequences ^3^
5	Issyk-Kul region, roadside stream (1 m)	42°26′48″ N 75°50′35″ E	1757	18 June	32:23	**-**
18	Naryn region, rocky roadside stream (1.5 m)	41°31′24″ N 75°02′10″ E	1912	21 June	10:0	-
20	Naryn region, meadow stream (1 m)	41°12′32″ N 74°45′42″ E	2080	22 June	20:4	-
21	Naryn region, polluted roadside stream (0.75 m)	41°13′54″ N 74°41′52″ E	1995	22 June	8:0	-
32	Naryn region, muddy roadside stream (2 m)	41°21′52″ N 73°44′40″ E	1827	23 June	55:11	3
34	Naryn region, roadside trickle (0.3 m)	41°19′17″ N 73°39′48″ E	2052	23 June	45:22	-
46	Jalal-Abad region, grassy mountainside stream (0.3 m)	40°33′05″ N 73°58′26″ E	1805	26 June	11:4	-
51	Osh region, Hwy. M41, 3 km W of Sary-Tash (3 m)	39°44′38″ N 73°12′25″ E	3270	28 June	1:1	-
52	Osh region, Hwy. A371, Tajikistan border, Kara-Kindik River (3–4 m)	39°42′00” N 73°27′16” E	3246	28 June	6:6	2
60	Osh region, Hwy. M41, Mashrapsay River (4–8 m)	40°17′09″ N 73°16′11″ E	2072	29 June	1:1	-
62	Osh region, wide valley stream (1 m)	40°16′01″ N 73°03′20″ E	1736	29 June	23:10	-
65	Jalal-Abad region, 0.25 km N of site 67 (1 m)	41°17′39″ N 72°40′39″ E	1762	1 July	34:23	3
67	Jalal-Abad region, ca. 3 km N of village of Alash (3 m)	41°17′32″ N 72°40′34″ E	1743	1 July	13:10	2
69	Jalal-Abad region, springfed stream with strong flow ca. 6 km NE of Maylisuu (0.15 m)	41°18′31″ N 72°29′51″ E	1098	2 July	21:7	2
75	Talas region, tumbling mountain stream ca. 8 km W of Toluk (1–3 m)	41°55′32″ N 73°28′45″ E	1870	3 July	8:5	2
78	Naryn region, roadside meadow trickle (0.15 m)	41°52′46″ N 73°41′43″ E	2348	4 July	10:5	-
85	Naryn region, Kekemeren River valley, tumbling stream ca. 2 km S of Kyzyl-Oi (10–15 m)	41°55′52″ N 74°09′11″ E	1737	5 July	2:2	-
88	Naryn region, roadside stream, ca. 5 km NW of Dzholkara (1.5 m)	41°53′15″ N 74°39′46″ E	2185	5 July	36:18	4
89	Naryn region, roadside stream ca. 1 km S of Bashkaingdy (15–20 m)	41°59′13″ N 74°39′41″ E	1804	5 July	4:4	-
95	Naryn region, Hwy. A367, roadside stream, ca. 6 km W of Uzunbulak (0.5 m)	42°06′07″ N 75°11′02″ E	2512	6 July	1:1	-

^1^ Distances are along straight lines. ^2^ Number of larvae collected into 1:3 acetic ethanol fixative and the number prepared chromosomally. Banding patterns from the following prepared larvae could not be completely interpreted due to poor chromosomal quality and are not included in the analyses or Table 2: site 5 (one larva), site 32 (four larvae), site 46 (one larva), site 62 (four larvae), site 65 (one larva), site 69 (five larvae), site 75 (one larva), and site 88 (one larva). ^3^ A hyphen (-) indicates that no larvae were sequenced.

**Table 2:** Frequency of all rearrangements in chromosomal constituents relative to the *Simulium* subgeneric banding sequence and frequency of B chromosomes for larvae of the *Simulium ornatum* group collected in Kyrgyzstan, June–July 2015.

Site	5	20	32	34	46	51	52	60	62	65	67	69	75	78	85	88	89	95
Females: Males	10:12	3 ^1^:1	5:2	12:10	3 ^2^:0	1:0	5:1	1:0	4 ^1^:2	12:10	7 ^1^:3	0:2	1:3	2:3	1:1	9:8	2:2	0:1 ^2^
IL-4	-	-	-	-	-	-	-	-	-	-	-	-	-	-	-	0.35	-	0.50
*IL-11*	1.00	1.00	1.00	1.00	1.00	1.00	1.00	1.00	1.00	1.00	1.00	1.00	1.00	1.00	1.00	1.00	1.00	1.00
IL-68	-	-	-	0.02	-	-	-	-	-	-	-	-	-	-	-	-	-	-
IL-69	-	-	-	-	-	-	-	-	-	-	-	-	-	-	-	0.03	-	-
IIL-19	-	-	-	-	-	-	-	-	-	-	-	-	-	0.10	-	-	-	-
IIL 2°NO ^3^	-	-	-	-	-	-	-	-	-	0.02	-	-	-	-	-	-	-	-
IIIS-3	-	-	-	* ^4^	-	-	-	-	-	* ^4^	* ^4^	* ^4^	* ^4^	-	-	-	-	-
IIIS-4	-	-	-	-	-	-	-	-	-	-	-	-	-	-	0.25	-	-	-
IIIS-5 ^5^	0.02	-	-	-	-	-	-	-	-	-	-	-	-	-	-	0.03	-	-
IIIS hb81	-	-	** ^6^	** ^6^	-	-	-	-	** ^6^	** ^6^	** ^6^	-	-	-	-	-	-	-
IIIS de79	-	-	-	-	-	-	-	0.50	-	-	-	-	-	-	-	-	-	-
IIIS hb73(1) ^5^	0.02	-	-	-	-	-	-	-	-	-	-	-	-	-	-	0.03	-	-
IIIS hb73(2) ^5^	0.02	-	-	-	-	-	-	-	-	-	-	-	-	-	-	0.03	-	-
IIIS hb74 ^5^	0.02	-	-	-	-	-	-	-	-	-	-	-	-	-	-	0.03	-	-
IIIS hb telo ^5^	0.02	-	-	-	-	-	-	-	-	-	-	-	-	-	-	0.12	-	-
*IIIL-1*	1.00	1.00	1.00	1.00	1.00	1.00	1.00	1.00	1.00	1.00	1.00	1.00	1.00	1.00	1.00	1.00	1.00	1.00
IIIL-39	-	-	0.07	-	-	-	-	-	-	-	-	-	-	-	-	-	-	-
IIIL-40	-	-	-	0.02	-	-	-	-	-	-	-	-	-	-	-	-	-	-
IIIL-41	-	-	-	-	-	-	-	-	-	-	-	-	-	-	-	0.09	-	-
IIIL-42	0.02	-	-	-	-	-	-	-	-	-	-	-	-	-	-	-	-	-
IIIL-43 ^7^	0.02	-	-	-	-	-	-	-	-	-	-	-	-	-	-	-	-	-
IIIL hb100 + telo ^8^	0.11	-	-	-	-	-	-	-	0.08	0.02	0.10	-	-	-	-	-	-	-
Bs (%)	-	-	-	-	-	-	0.33	-	-	-	-	-	-	-	-	-	-	-

Note: Cells with a hyphen indicate 0.00 values. ^1^ Larvae were infected with unidentified mermithid nematodes as follows: site 20 (three females), site 62 (one female), and site 67 (one female). ^2^ Larvae were infected with a microsporidian-like pathogen in the fat body but no spores were present, as follows: site 46 (one female) and site 95 (one male). ^3^ A secondary nucleolar organizer was expressed heterozygously in section 62. ^4^ * indicates that IIIS-3 was linked to the Y chromosome (Y_1_) and was heterozygous in the following fifteen male larvae: three (site 34), eight (site 65), one (site 67), two (site 69), and one (site 75); one female at site 67 was heterozygous for IIIS-3; and all other males and females were standard for IIIS-3. ^5^ IIIS-5, IIIS hb73(1), IIIS hb73(2), IIIS hb74, and IIIS hb telo were found on the same homologue in one male larva at each of sites of 5 and 88. The telomeric region of IIIS expressed strong heterochromatinization with either flaring or compaction in the same larva at site 5. Two female larvae at site 88 also were heterozygous for IIIS hb telo (flared) but did not carry inversions or band amplifications. ^6^ ** indicates that IIIS hb81 was linked to the X chromosomes (X_1_) in the following female larvae: heterozygous in two (site 32), three (site 34), two (site 62), and two (site 65); it was homozygous in one (site 67). ^7^ IIIL-43 was on the same homologue as IIIL hb100 + telo in one male. ^8^ The telomeric region of IIIL expressed a strong heterochromatic band and a slightly enhanced subterminal band (hb) in section 100.

## Data Availability

All data supporting the reported results are included in the text and, for molecular data, also in GenBank.
